# Refining Lung Cancer Brain Metastasis Models for Spatiotemporal Dynamic Research and Personalized Therapy

**DOI:** 10.3390/cancers17091588

**Published:** 2025-05-07

**Authors:** Ying Chen, Ao Zhang, Jingrong Wang, Hudan Pan, Liang Liu, Runze Li

**Affiliations:** 1 Chinese Medicine Guangdong Laboratory, The Second Affiliated Hospital of Guangzhou University of Chinese Medicine, Guangzhou 510006, China; 20221120321@stu.gzucm.edu.cn (Y.C.); 20242110195@stu.gzucm.edu.cn (A.Z.); jrwang@gzucm.edu.cn (J.W.); hdpan@gzucm.edu.cn (H.P.); 2 School of Pharmaceutical Sciences, Guangzhou University of Chinese Medicine, Guangzhou 511430, China

**Keywords:** non-small cell lung cancer, brain metastasis, animal models, translational oncology, drug delivery

## Abstract

Lung cancer brain metastasis (LCBM) is a major contributor to cancer-related mortality, with a median survival of 8–16 months following diagnosis, despite advances in therapeutic strategies. The development of clinically relevant animal models is crucial for understanding the metastatic cascade and assessing therapies that can penetrate the blood–brain barrier (BBB). This review critically evaluates five primary LCBM modeling approaches—orthotopic implantation, intracardiac injection, stereotactic intracranial injection, carotid artery injection, and tail vein injection—focusing on their clinical applicability. We systematically compare their ability to replicate human metastatic pathophysiology and highlight emerging technologies for personalized therapy screening. Additionally, we analyze breakthrough strategies in central nervous system (CNS)-targeted drug delivery, including microparticle targeted delivery systems designed to enhance brain accumulation. By incorporating advances in single-cell omics and AI-driven metastasis prediction, this work provides a roadmap for the next generation of LCBM models, aimed at bridging preclinical and clinical research.

## Simple Summary

This study provides a comprehensive review of lung cancer brain metastasis (LCBM) models, evaluating their clinical relevance and ability to replicate human metastatic pathophysiology. The authors assess five primary modeling approaches—orthotopic implantation, intracardiac injection, stereotactic intracranial injection, carotid artery injection, and tail vein injection—highlighting their strengths and limitations. This review emphasizes the need for robust models that can accurately simulate the metastatic cascade and aid in developing therapies capable of penetrating the blood–brain barrier (BBB). This article also explores emerging technologies in personalized therapy screening and CNS-targeted drug delivery, aiming to bridge preclinical and clinical research.

## 1. Introduction

Lung cancer accounts for 12.4% of global cancer incidence and 18.7% of cancer-related mortality, with BMs occurring in 25–65% of patients [1]. Prognosis remains poor, with a median survival of 1–2 months without treatment [2]. Tumor heterogeneity, BBB impermeability, and drug resistance contribute to therapeutic failure, highlighting the need for robust animal models that accurately replicate human LCBM pathophysiology. This review evaluates current methodologies for developing LCBM models, their clinical relevance, and their applications in mechanistic and therapeutic research.

## 2. Biology of Lung Cancer Brain Metastases

The interaction between LCBM and the tumor microenvironment (TME) is critical to understanding its complex pathophysiology. This section examines the biological mechanisms of LCBM through three key processes: (1) BBB transmigration, (2) cerebral colonization, and (3) TME-mediated progression.

### 2.1. Mechanisms of Lung Cancer Brain Metastases

**Figure 1:**
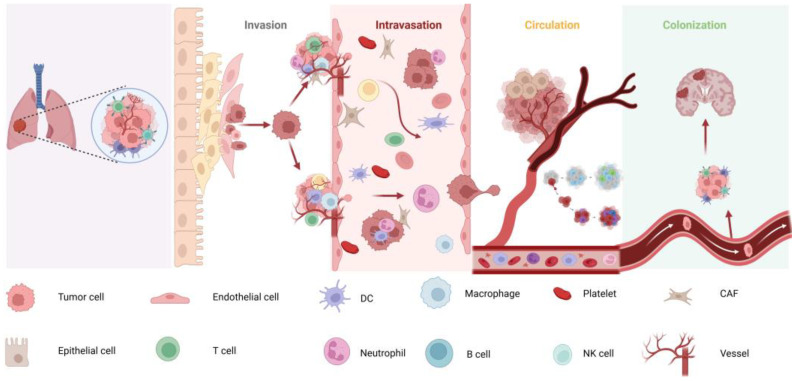
Schematic of LCBM mechanism.

Tumor metastasis is a multistep process—comprising invasion, intravasation, circulation, and colonization (Figure 1) [3,4]. The brain’s extensive vascular network and its proximity to the pulmonary circulation make it a common metastatic site for lung cancer [5]. Although tumor cells utilize various pathways to infiltrate the CNS, the precise molecular drivers remain incompletely understood [6]. A key therapeutic challenge is the BBB, which limits drug penetration and complicates treatment efficacy [7].

Qian Huang et al. [8] demonstrated that perivascular cells derived from CD44+ lung cancer stem cells in lung adenocarcinoma promote brain metastasis through transendothelial migration, a process enhanced by G protein-coupled receptor 124 (GPR124). Shengkai Xia et al. [6] revealed that mesothelin plays a critical role in BMs in non-small cell lung cancer (NSCLC) via the JNK/MET signaling pathway, highlighting it as a potential therapeutic target for preventing brain metastasis in NSCLC patients. Jung Eun Lee et al. [9] found that lung cancer cells undergo mesenchymal–epithelial transition during brain metastasis colonization. Furthermore, they showed that Noggin knockdown inhibits the migration and invasion of lung cancer cells during this process.

### 2.2. Impact of the TME on BM

The TME plays a multifaceted role in brain metastasis. It not only provides suitable growth conditions for tumor cells but also promotes tumor invasion and metastasis through various mechanisms [10]. Key components of the TME, such as the extracellular matrix (ECM), immune cells, vascular endothelial cells, and tumor-associated fibroblasts (CAFs), release a range of growth factors, cytokines, and chemokines, collectively shaping a microenvironment that supports tumor growth and metastasis [11]. Additionally, immune cells within the TME, such as tumor-associated macrophages (TAMs) and myeloid-derived suppressor cells (MDSCs), suppress anti-tumor immune responses by releasing immunosuppressive molecules, thereby facilitating tumor cell escape and metastasis [12]. The TME further promotes tumor invasion and metastasis by inducing epithelial–mesenchymal transition and angiogenesis [13]. These complex interactions and signaling pathways contribute to the pro-tumorigenic effects of the brain metastasis microenvironment, creating favorable conditions for tumor cell colonization and growth. Therefore, a comprehensive understanding of the TME’s role in brain metastasis is essential for the development of novel therapeutic strategies.

### 2.3. The Role of the BBB in Metastasis

The BBB is a highly selective barrier composed of capillary endothelial cells, basement membranes, astrocytic end-feet, pericytes, and other components in the brain. Its primary function is to protect brain tissue from harmful substances while impeding the passage of tumor cells [14]. Lung cancer cells can cross the BBB through the following mechanisms: (1) Destruction of BBB structure: Tumor cells secrete protein hydrolases, such as matrix metalloproteinases (MMPs), which degrade the basement membrane and tight junction proteins of the BBB, increasing its permeability [15]. (2) Cell–cell adhesion and migration: Lung cancer cells adhere to cerebrovascular endothelial cells via specific adhesion molecules (e.g., integrins and selectins) and cross the BBB through pseudopod extension and cell migration [16]. (3) Role of extracellular vesicles: Lung cancer cells release extracellular vesicles, such as exosomes, which carry proteins and nucleic acids that modulate BBB permeability and facilitate tumor cell crossing [17,18].

## 3. Classification of Model Sources

As elucidated in the foregoing content, LCBM represents a highly intricate biological process. A profound comprehension of this process is of utmost significance, and the establishment of animal models capable of precisely mimicking human LCBM is essential. Such models serve a dual-purpose role: they facilitate the exploration of the molecular mechanisms underlying LCBM and lay a crucial experimental foundation for the development of novel therapeutic strategies. Consequently, the selection of suitable experimental animals emerges as a pivotal step in the construction of effective models. An ideal LCBM animal model should replicate the entire process of lung cancer, including initiation, progression, invasion, and metastasis to the brain. It should also possess characteristics such as easy availability, simple handling, good reproducibility, histological and biological similarity to human tumors, and a high success rate in transplantation [19]. Mice are commonly used in scientific research due to their ease of maintenance, high reproductive rate, and versatility. LCBM models are typically divided into models using immunologically intact mice and immunodeficient mice.

### 3.1. Immunologically Sound Mice

Common mouse strains with normal immune function include C57BL/6 and BALB/c mice. C57BL/6 mice offer advantages such as normal immune function, low cost, high tolerance, and well-established models, making them commonly used for constructing lung cancer metastasis models using Lewis cells. BALB/c mice, which are highly fertile and have a longer breeding cycle, are generally social and easy to house in groups.

### 3.2. Immunodeficient Mice

Immunodeficient mice, such as nude mice and Severe Combined Immunodeficiency (SCID) mice, are frequently used for tumor studies. Nude mice are hairless mutants with a congenital absence of the thymus, resulting in thymus-dependent immune system deficiency and minimal immune rejection. These mice can be used in tumor transplantation models without requiring special pre-treatment [20]. SCID mice lack mature T and B cell immunity due to an autosomal recessive mutation, and human tumor transplant survival rates are generally higher in SCID mice than in nude mice. These mice are particularly useful for studying human tumor transplantation and simulating the human tumor microenvironment. However, over time, SCID mice may gradually produce mature T and B cells (a phenomenon known as “leakage”), which can reduce the accuracy of experiments [21,22].

### 3.3. Source of Transplanted Cells

LCBM transplantation models are categorized based on the source of transplanted cells: animal-derived and human-derived models. Animal-derived models have the advantage of preserving immune function, allowing for the study of host–tumor cell interactions, and having a shorter modeling cycle. These models are typically used in immunologically intact animals. However, they have limitations, such as biological differences between animal-origin tumor cells and human patient-derived tumor cells, as well as lower rates of brain parenchymal metastasis [23,24]. In contrast, human-derived models more accurately replicate the characteristics of patient tumors, preserving tumor heterogeneity and providing more reliable results for drug efficacy assessment, drug screening, and clinical trials. The main disadvantage of human-derived models is that they require immunodeficient animals and lack normal immune interactions with tumor tissues.

## 4. Methods of Constructing BMs Models for Lung Cancer

**Figure 2:**
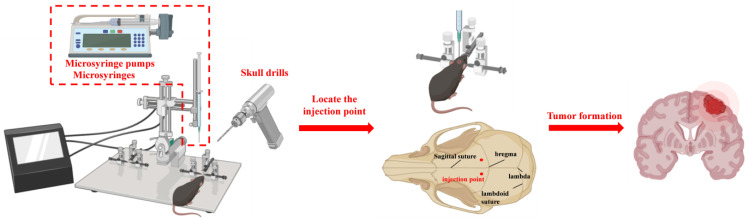
The main procedures of stereotactic injection in the brain.

**Figure 3:**
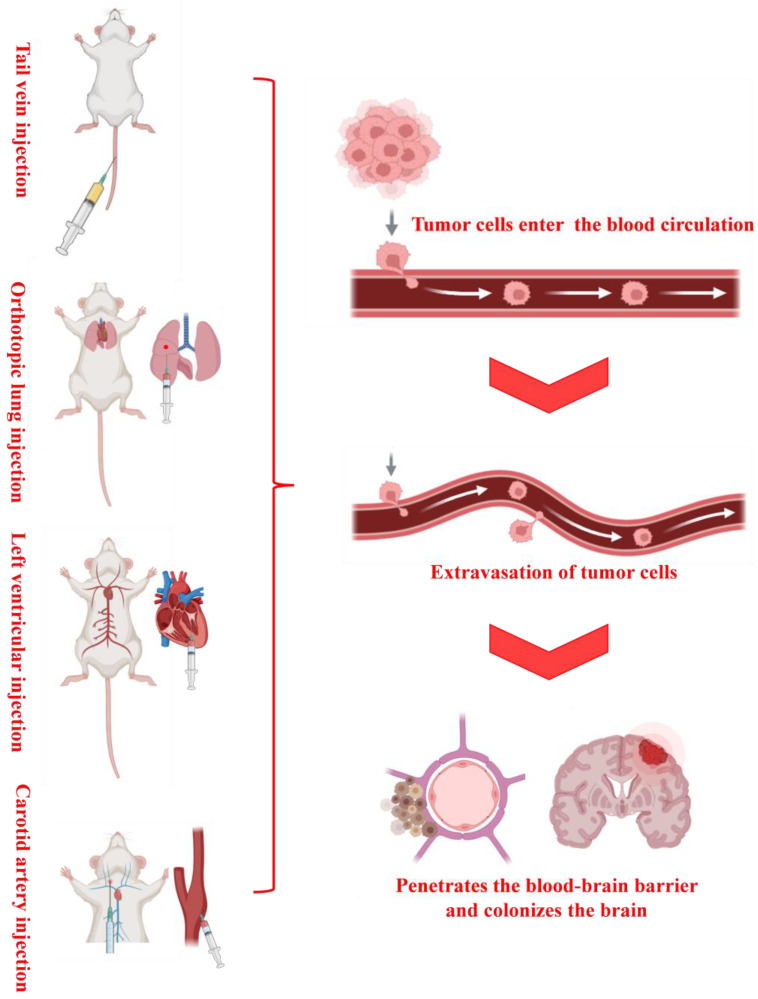
Sketch of the four modeling methods and tumor cell transfer and colonization.

In practical research, diverse experimental requirements and research objectives dictate the choice of model construction methods. Each method possesses its distinct advantages and disadvantages, rendering it suitable for different research scenarios (Table 1 and Table 2). Therefore, the selection of an appropriate model construction method is of paramount importance for ensuring the reliability and validity of experimental results. Currently, common methods for constructing animal models of LCBM include five main approaches: brain stereotaxic injection [25,26,27], caudal vein injection [28,29], carotid artery injection [30,31], left ventricle injection [6,32], and in situ injection [33] (Figure 2 and Figure 3); these methods each have their unique application scenarios and key operation points, which will be discussed in detail in the subsequent sections.

### 4.1. Stereotactic Intracranial Injection

Intracerebral injection is a relatively simple and efficient modeling method, often assisted by a small animal stereotactic frame or a brain stereotactic apparatus. It is a fast procedure with a high success rate, making it particularly suitable for tumor cell lines that do not have obvious brain metastatic characteristics. However, because the cancer cells are not directly implanted into the mouse brain, the metastatic cascade is often overlooked, which limits the ability to simulate the full tumorigenesis process [34].

Steps for intracerebral injection [35] (Figure 2): Mice are anesthetized and secured in a brain stereotactic apparatus. The skin is prepared and disinfected, and a small incision (approximately 1 cm) is made along the sagittal suture to expose the cranial bone. The periosteum is pushed aside to expose the fontanel, which serves as the reference point. From there, the point 2 mm laterally and 0.5 mm anterior to the fontanel is located to target the striatum. A small circular hole is drilled in the cranial bone using a drilling needle. Next, a micro-injector is used to aspirate 5 μL of the cell suspension to a depth of approximately 3 mm. The suspension is slowly injected into the striatum at a uniform speed. After injection, the needle is left in place for 3 min, then withdrawn slowly. The hole is sealed with bone wax, the skin is disinfected, and the incision is sutured.

Ozama et al. [27] used straightedge assistance to precisely locate the injection site, achieving a modeling rate of up to 30 mice injected per hour. They successfully established an intracranial transplantation tumor model in nude mice, with 5 × 10^4^ cells injected intracranially per mouse. The nude mice survived for several months, allowing for a prolonged experimental period. Lockman et al. [36] injected Lewis lung cancer cells into the right hemisphere of BALB/c mice using brain stereotaxic injection to assess BBB permeability and the expression of P-glycoprotein (P-gp) at various stages of brain tumor development. Their results showed that as the tumor developed, BBB permeability increased while P-gp expression decreased, which could potentially affect the efficacy of chemotherapeutic agents.

### 4.2. Tail Vein Injection

Tail vein injection is a relatively simple technique compared to other modeling methods; however, most tumor cells tend to be retained in the lungs after tail vein injection, leading to a lower likelihood of brain metastases [31]. Moreover, the malignancy of lung cancer often causes the mice to die before brain metastases can form. Additionally, intravenous injection does not fully replicate the clinical process, as metastases are primarily transported via arterial blood in patients.

The tail vein injection procedure involves mounting the mice in a restrainer with a tight lid, ensuring that the tail faces outward. The tail is cleaned with an alcohol swab to dilate the veins, then held in place using the thumb, index, and middle fingers to straighten it. The visible red vein is the target for injection. A 1 mL syringe (100 μL, 1 × 10^6^ cells) is used, with the needle inserted at a 15° angle, approximately 1/3 of the way from the rear of the tail. If the needle meets no resistance, it is correctly positioned in the vein, and injection can proceed. If there is resistance or bulging, it indicates subcutaneous injection, and the syringe should be withdrawn and re-inserted slightly above the original site. After injection, a cotton ball is applied to the injection site for 1 min to stop bleeding. Once bleeding ceases, the incision is closed and the mouse is removed from the restrainer [37].

### 4.3. In Situ Injection

In situ injection methods include both trans-chest wall and trans-lung lobe injection. The trans-chest wall injection method is simpler and avoids the need to open the chest, resulting in a lower mortality rate, though it does not allow for precise control over the injection site. In contrast, in situ inoculation models are the most representative of clinical brain metastasis formation, as they encompass all stages of metastatic development. However, early studies indicated that in situ inoculation of tumor cells may be less effective in forming metastatic foci, particularly in later clinical stages. Recent advancements in cell lines with enhanced metastatic properties, genetic engineering to modify the inoculation microenvironment, and other techniques have improved the efficacy of in situ models. Due to anatomical considerations and clinical relevance, in situ models are often used in studies on lung cancer, breast cancer, and melanoma. For instance, human small cell lung cancer cell lines, such as NCI-H187 and DMS273, inoculated into nude mouse lungs can lead to metastasis in the bone, kidney, and brain [38].

For the main operation procedure of trans-thoracic wall injection [33], after anesthesia, the mice were fixed in the right lateral recumbent position, the skin was prepared, the left chest skin was disinfected, and the insulin needle was used to aspirate 0.2 mL of cell suspension, and the needle was inserted in the 6th intercostal space of the posterior axillary line, and attention was paid to the control of the depth of the needle insertion in the process of the injection (subject to the sense of the needle breakthrough before inserting the needle for another 3–4 mm). For the main operation steps of the translobar injection [39], after anesthesia, the mice were fixed in the right lateral position, the mice were prepared for the axillary skin, disinfected, and an incision of 5 mm size was made in the left anterior axillary line at about 1.5 cm above the rib arch, separating the skin and subcutaneous tissues, exposing the thoracic wall, and then the lobes of the lungs were seen to be moving up and down with the respiration, and then 50 μL of the cell suspension was sucked up with the microfeeder, and then injected to the left lung, and the depth of injection was about 3 mm, and then stopped for a few seconds after injection. The needle was injected into the left lung at a depth of about 3 mm, the needle was stopped for a few seconds after injection, and the incision was closed after the needle was withdrawn.

**Table 2:** Comparative analysis of LCBM animal model construction methods.

Modeling Method	Metastatic Cascade Replication	Clinical Relevance	Technical Difficulty	Immune Compatibility	Metastasis Rate (%)	Key Applications	Refs.
Stereotactic Intracranial Injection	Partial (skips BBB transmigration)	Low	Low	Immunodeficient	90–100	Pharmacological drug testing	[40,41]
Tail Vein Injection	Partial (lung retention bias)	Moderate	Low	Immunocompetent/Deficient	5–15	Metastatic seeding studies	[42,43,44]
Carotid Artery Injection	High (hematogenous spread)	High	High	Immunocompetent	70–85	BBB transmigration studies	[31,45]
Left Ventricular Injection	High (systemic circulation)	High	Moderate	Immunodeficient	60–75	Systemic metastasis dynamics	[46,47]
Orthotopic Implantation	Full (primary tumor progression)	Very High	High	Immunocompetent	20–40	Tumor-microenvironment interactions	[48,49,50]

### 4.4. Left Ventricular Injection

Left ventricular injection is a commonly used method for constructing brain metastasis models, as it simulates the hematogenous spread of tumors seen in clinical settings. However, this technique poses challenges, such as the inability to visualize the injection site in blind procedures, leading to potential errors like the formation of metastases near the injection site. Using small-animal ultrasound imaging can enhance accuracy and increase the success rate.

For the main operation steps of intracardiac blind injection in mice [51,52], mice were fixed in the supine position after appropriate anesthesia, and the chest was disinfected and prepared for skin, the number of tumor cells injected was about 1 × 10^6^ cells, the injection was performed with an insulin syringe, the injection point was marked at the left of the midpoint of the upper sternal notch and the raphe, and the needle could be inserted vertically from the left ventricular cavity, the injection point could also be moved outward and downward a little bit, and the needle was inserted toward the left ventricle at an angle of 45° to facilitate the use of the injection propulsion device, when blood jet was seen pouring in to indicate that the needle entered the left ventricle; after injection, the needle was slowly withdrawn with slight negative pressure. In order to facilitate the use of the injection propulsion device, when you see the blood jet influx indicates that the needle enters the left ventricle, immediately fix the position of the needle, and slowly inject the tumor cells into the left ventricle; after the injection is completed, back out of the needle with a slight negative pressure. For the main operation steps of small animal high-resolution ultrasound imaging system-assisted left ventricular injection [53], after the animals were anesthetized and fixed in the supine position, the position of the ultrasound imaging probe was adjusted to clearly display the echocardiographic image of the left ventricle, then the syringe was fixed to the manipulable advancement device, and the tip of the needle was advanced into the cavity of the left ventricle with the assistance of dynamic ultrasound imaging for accurate injection. However, even if the intracardiac injection is completed accurately, a portion of the modeled animals will still die after the injection of tumor cells because tumor cells have procoagulant activity, which can cause extensive thrombosis and embolism, thus leading to the death of the animals. Stocking et al. [54] showed that low-molecular-weight heparin (10 mg/kg) injected through the tail vein 10 min before transmural inoculation of tumor cells into the left ventricle greatly reduced inoculation-induced mortality in modeled animals.

Jin Yu et al. [55] screened the transfected luciferase lung cancer cell line A549-F3 with brain metastatic properties by in vivo cycling and then constructed a brain metastasis model by left ventricular injection (2 × 10^5^ cells) to study the mechanism of brain metastasis occurrence, and at the endpoint of the experiments, the mice were imaged in vivo for calculating the rate of brain metastasis, and the success rate of modeling was 66.7% (8/12). Uzunalli et al. [56] used lentivirus to infect A549 cells to make them stably express luciferase and then used ultrasound-induced intracardiac injection to inoculate A549 cells (1 × 10^6^ cells) into nude mice, and 2 weeks after the inoculation, the pathological examination showed that tiny metastatic foci appeared in the brain of nude mice.

### 4.5. Carotid Injection

Carotid artery injection involves isolating the common carotid artery through a longitudinal neck incision, puncturing it, and injecting tumor cells. This method effectively simulates hematogenous brain metastasis but requires precise technique and is technically challenging. Though the injection site is visible, finding and accurately targeting the carotid artery is difficult. The technique demands careful control of the injected tumor cell volume and number.

In mouse carotid artery injection [57], mice were anesthetized and fixed in the supine position, the skin was prepared and sterilized, and a 1 cm median incision was made in the neck, and the muscle and carotid sheath was bluntly separated along the left/right side downward to isolate the common carotid artery of the side, carefully isolating the vagus nerve near the common carotid artery and minimizing bleeding. After isolating one side of the common carotid artery, use 6–0 sutures to ligate the external carotid artery at the bifurcation of the proximal common carotid artery and the distal end, and tie a loose knot at the distal end of the common carotid artery for later use, and use a 32G insulin syringe to aspirate the cell suspension, taking care to avoid inhaling air bubbles. The number of injected aneurysmal cells was usually around 5 × 10^4^ cells, and the needle was inserted at 5–6 mm below the bifurcation of the common carotid artery, injected at a uniform rate within 1 min. After injection, a gelatin sponge was used to gently press or silk thread ligation was used to stop bleeding, the incision was sutured, sterilized with iodine, and placed on a heating pad to maintain the body temperature, and then returned to the rat box after anesthesia resuscitation; the whole operation process should be controlled within 15 min as much as possible

Zean Zhang et al. [30] used an optimized carotid artery injection method by injecting Lewis lung cancer cells into the internal carotid artery of C57BL/6J mice and tightening the injection point after the completion of the injection, and brain metastasis was detected in all five mice at 15 days after the operation. Researchers Chen Yan et al. [58] constructed a brain metastasis model by injecting PC-9 cells into the internal carotid arteries of nude mice, which were used for the in vivo pharmacokinetics and pharmacodynamics evaluation. For in vivo pharmacokinetic and pharmacodynamic evaluation, it was found that the concentration of gefitinib in blood, brain, and cerebrospinal fluid showed a dose-dependent increase in both normal mice and brain metastasis model mice.

Overall, although these models have preliminarily established an experimental basis for simulating lung cancer brain metastasis, further research and optimization of the models are essential to more accurately reveal its pathogenesis and evaluate treatment strategies. In terms of model optimization, Zihao Liu et al. [59] constructed a novel optimized orthotopic mouse model for lung cancer brain metastasis (OOMM) to address the shortcomings of traditional models. This model utilized a light controlled GelMA/HAMA hydrogel to repair the puncture site of the carotid artery, maintaining cerebral blood circulation and overcoming the defect of permanent carotid artery ligation in traditional models. In the experiment, the researchers characterized the synthesized hydrogel, obtained labeled cells through cell culture and stable transfection, and compared various indicators of the traditional model (TOMM) and OOMM in animal experiments. The results showed that the OOMM significantly improved cerebral blood flow and oxygen supply, reduced the degree of cerebral ischemia and hypoxia, increased the postoperative survival rate of mice and the success rate of brain metastasis, increased the number of tumor clones, and altered the spatial distribution of tumors in the brain. Li Wang et al. [60] developed an optimized model protocol for screening lung cancer cell lines with high brain metastatic tropism through repeated intracardiac injections in mice to improve the deficiencies of traditional models in simulating the lung cancer brain metastasis process. This protocol used a multi-round in vivo screening method and detailed the experimental steps from inoculating tumor cells to gradually isolating, culturing, and screening brain metastatic subpopulations, overcoming the defects that traditional models could not accurately simulate the natural metastasis of tumor cells in the body and screen for cells with high metastatic characteristics.

In the key progress and breakthroughs in lung cancer brain metastasis research, Hao Duan et al. [61] conducted multiomics integration analysis of the primary tumors and brain metastatic lesions of 154 lung cancer patients. Through whole genome sequencing, transcriptome sequencing, and metabolome analysis, they revealed the mutation characteristics and metabolic differences between primary lung cancer and brain metastatic lesions. The study found that the mitochondrial metabolism of brain metastatic lesions was active, especially the abnormal oxidative phosphorylation pathway. Based on this, they used the carotid artery injection model for verification and found that the drug Gamitrinib targeting this pathway could induce apoptosis and inhibit the proliferation of brain metastatic tumor cells. Moreover, the combined use of oxidative phosphorylation inhibitors and anti-PD1 immunotherapy significantly prolonged the survival of mouse models of lung cancer brain metastasis. Minjie Fu et al. [45] performed single cell sequencing on lung cancer brain metastatic samples and found that tyrosine kinase inhibitors could reshape the immune microenvironment of lung cancer brain metastasis during treatment. While increasing T cell infiltration, they caused high expression of the immune checkpoint CTLA4, leading to immune escape. To explore effective countermeasures, they combined two models, brain stereotactic injection and left ventricular injection, and verified in animals that combined targeted therapy and CTLA4 monoclonal antibody could effectively activate T cells and overcome immune escape. These studies not only optimized the animal models of lung cancer brain metastasis but also deeply revealed the key mechanisms of lung cancer brain metastasis. This series of advancements brings new hope for the innovation and development of treatment strategies for lung cancer brain metastasis and is expected to promote substantial breakthroughs in this field.

## 5. Brain Metastasis Modeling Assays

After successfully establishing a model of lung cancer brain metastasis, accurately evaluating the biological characteristics and metastatic ability of the model is a crucial step in validating its effectiveness and reliability. The detection methods for brain metastasis tumor models can not only assist us in understanding the growth, invasion, and metastasis of tumors but also provide key data for the subsequent evaluation of treatment strategies. Therefore, the selection of appropriate detection methods is of vital importance for ensuring the accuracy and reliability of research results. With the continuous development of science and technology, breakthroughs in brain metastasis detection methods are also being made. In the future, we expect that more progress is expected in the following directions:

### 5.1. Imaging Tests

Regular imaging tests for brain metastases can provide information on the continuous dynamic development of multiple lesions. Clinical brain metastases are examined by CT, positron emission imaging (PET), magnetic resonance imaging (MRI), small animal biopsy, etc. Currently, MRI and small animal biopsy techniques are mostly used [62,63].

Early 3.0T MRI was mostly used in larger animal models such as nude rat models, and the detection sensitivity for nude mice was insufficient; the latter required the application of a small animal MRI machine of 7.0T or above to further improve the accuracy and clarity. Engin Dikici et al. [64] found that the coil MRI scanning of small animals could clearly detect brain metastases, especially metastases of deeper and smaller brain tissues However, due to the technical problems of the equipment such as resolution, the detection of early tiny metastases is still difficult. Moreover, this method cannot estimate the size of tumor lesions, and can only be used for preliminary localization.

Small animal in vivo imaging techniques include fluorescence and bioluminescence: fluorescence luminescence requires an exogenous excitation light to make the fluorescent moiety GFP emit longer wavelength emission light, but there are a variety of substances in the organism that can emit non-specific fluorescence under the effect of excitation light, which affects the detection of the sensitivity, whereas bioluminescence imaging is more sensitive compared to fluorescence imaging and does not require excitation light, but only the use of the luciferase reporter gene tags tumor cells to stably express luciferase, which converts chemical energy into light energy under the action of luciferin substrate, oxygen, Mg^2+^, and ATP, and the animal itself does not emit light [65]. It has been suggested that [66] fluorescence luminescence technology is suitable for imaging studies of tumors at superficial sites or at the cellular level in vitro, whereas bioluminescence imaging is suitable for the study of deep tissue primary or metastatic tumors. Some researchers [67] inoculated Lewis cells transfected with reporter genes into mice and injected 150 mg/kg of bioluminescent substrate intraperitoneally 15 min before the test from day 2 onwards; anesthetized mice underwent small-animal in vivo imaging to dynamically detect the tumor size and an exponential growth of tumors could be observed, which is similar to the results of HE staining. Small-animal in vivo imaging has many advantages, such as the following: can be repeated in the same body many times to obtain a series of data, eliminating individual differences; can be dynamic observation of the results of the experiments, Fetting intuitive images, the results at a glance; can be non-invasive detection of specific biological behaviors in vivo, and maximize the simulation of the physiological and pathological state in vivo [68].

### 5.2. Pathological Testing

#### 5.2.1. Hematoxylin-Eosin (HE) Staining

In the field of tumor research, in-depth analysis of tumor tissues is the key link to understanding the mechanism of tumor development and evaluating the effectiveness of treatment. As an important research tool, the most basic and widely used method is HE staining.

For the study of animal models of brain metastases, it is crucial to use a series of intact sections of the whole brain as much as possible in order to obtain comprehensive and accurate information. This is because the distribution of brain metastases in the brain may be more diffuse, and complete sections provide a more complete picture of the location, size, and relationship of the tumor to the surrounding brain tissue. The principle of HE staining is based on the specific affinity of two dyes, hematoxylin, and eosin, for the different components of tissue cells. Hematoxylin is a basic dye that stains chromatin in the nucleus and ribosomes in the cytoplasm a purplish-blue color, while eosin is an acidic dye that stains components of the cytoplasm and extracellular matrix a red color. With this staining method, researchers were able to clearly observe the degree of differentiation and the size of the heterogeneity of the tissue cells.

In the brain metastatic tumor model, although the morphological differences in tumor cells were relatively small compared with clinical specimens, HE staining still revealed distinctive features. The nuclei of tumor cells are usually darker in staining, which reflects their higher nucleic acid content and more active metabolism; the dense arrangement of the nuclei suggests that the cells are proliferating vigorously; at the same time, the phenomenon of nuclear division is easily seen, which further confirms the rapid growth characteristics of tumor cells [69]. However, this classical assay also has some limitations. As it is necessary to execute the animals to obtain tissue samples for staining analysis at a specific time point after the modeling is completed, this leads to the impossibility of conducting continuous dynamic studies on the same animal model. Continuous dynamic studies are important for observing tumor progression and evaluating the effects of therapeutic treatments at different stages of time, and the shortcomings of HE staining in this regard have prompted researchers to explore and develop new detection techniques and methods.

#### 5.2.2. Immunohistochemistry and Immunoblotting

As a key bridge connecting basic research and clinical application, the detection of molecular markers in tumor tissues can provide an indispensable basis for precise diagnosis, rational classification, prognosis assessment, and targeted therapy of tumors. Immunohistochemistry, with its high specificity and sensitivity, occupies a central position in the field of molecular marker detection of tumor tissues and has become an indispensable key technology for the promotion of precise diagnosis and research of tumors [70].

The basic principle of immunohistochemistry is to identify and localize target antigens, i.e., molecular markers, in tissue cells by labeling specific antibodies, using the specific binding reaction between antigens and antibodies. For example, some cellular structural proteins play an important role in determining the origin of a tumor. Cytokeratin is mainly expressed in epithelial and mesothelial cells, as well as in cancer cells, and when keratin expression is detected in tumor tissues, it strongly suggests that the tumor may originate in epithelial tissue [71]. Different types of keratins also have finer tissue specificity, which helps to further clarify the specific origin and differentiation of the tumor. Vimentin, on the other hand, is mainly found in mesenchymal tissues as well as sarcoma cells, and its expression in tumor tissues can serve as an important clue to determine whether the tumor originates from mesenchymal tissues or not [72].

Not only that, the commonly used clinical tumor molecular markers are also of great value in the corresponding animal models of brain metastases. For example, carcinoembryonic antigen (CEA) is often highly expressed in a variety of tumors such as gastrointestinal tumors, lung cancer, etc. Detecting the level of CEA in brain metastasis animal models can not only assist in determining whether the tumor has metastasized from the primary foci but also assess the developmental process of the tumor as well as therapeutic efficacy by monitoring the dynamic changes [73]. Prostate-specific antigen (PSA) is of great significance for the study of animal models of prostate cancer brain metastases, which can help researchers clarify the origin and nature of the tumor and provide key information for in-depth study of the mechanism of prostate cancer brain metastases [74].

Immunohistochemical detection of molecular markers in tumor tissues, whether at the level of cytoarchitectural proteins to determine the origin of tumors or at the level of clinically commonly used molecular markers to analyze the characteristics of tumors, provides rich and valuable information for tumor research, especially brain metastases, and greatly promotes the development of basic research and clinical treatment in the field of oncology.

#### 5.2.3. Biomarker Analysis

In recent years, with the development of molecular biology, many novel biomarkers have been successively discovered and studied in clinical specimens such as tumor tissues, peripheral blood, and cerebrospinal fluid of patients with brain metastases of lung cancer, while blood samples are more easily available in the clinic, so the biomarkers in the peripheral blood of brain metastases of lung cancer are now elaborated (Table 3).

The neurobiochemical marker S100B protein is well known as a more sensitive brain injury-specific marker. The current study suggests that the S100B protein may become an indicator for assessing disease changes, treatment effects, and prognosis in some malignant tumors, especially in patients with brain metastases [75]. The correlation between serum S100B protein and the prediction of brain metastasis in lung cancer has been reported in a study by Vogelbaum et al. [76]. They tested the serum S100B protein concentration and MRI scans in 38 newly diagnosed lung cancer patients without neurological symptoms and without a known history of brain metastasis. Obvious microvascular changes were 0.5 ± 0.28 μg/L in the group with obvious microvascular changes, and 0.28 ± 0.19 μg/L in the group with brain metastasis on MRI scanning, suggesting that serum S100B can be used as a tool for predicting or detecting brain metastasis.

Myelin basic protein (MBP) is a protein specific to the CNS. After brain metastasis of lung cancer, damage to the CNS is caused by invasion and compression of cancerous tissues, which in turn causes an increase in the level of peripheral blood MBP. Studies have shown [77] that the serum level of MBP in the LCBM group was significantly higher than that in the lung cancer group, benign lung disease group, and healthy control group, and the difference between the lung cancer group and the benign lung disease group and the healthy control group was not statistically significant, and the serum level of MBP was independent of the size of the tumor. It is suggested that serum MBP has high specificity in the diagnosis of brain metastasis of lung cancer, and MBP can be used as a serological marker for early diagnosis of brain metastasis of lung cancer.

Cluster of differentiation 44 variant 6 (CD44V6): CD44 gene is a metastasis suppressor gene localized in llpl3, and encodes the standard CD44S and variant CD44V according to the difference in the expression of an exon. CD44 is closely related to tumor invasion and metastasis and is considered to be a tumor metastasis-related gene, among which the CD44 variant containing exon V6 is most closely related to tumor invasion and metastasis. It is considered a tumor metastasis-related gene, with the CD44 variant containing exon V6 being the most closely related to tumor invasion and metastasis. Studies have shown [78] that the expression of CD44V6 in cancer tissues of lung cancer patients with brain metastasis is significantly higher than that in cancer tissues of lung cancer patients without brain metastasis, suggesting that it is of great significance in predicting the trend of brain metastasis and judging the prognosis of patients.

E-cadherin (E-cad) is a calcium-dependent transmembrane glycoprotein widely expressed in various types of epithelial cells, and its lack of expression in tumor cells can reduce the homogeneous adhesion between cells, promote tumor proliferation and metastasis, and affect the prognosis of patients. Studies have shown [79] that the expression of E-cad in tumor tissues of patients with LCBM is significantly lower than that in tumor tissues and paracancerous tissues of patients with lung cancer without brain metastasis, and this mechanism may be the hypermethylation of the promoter region of the E-cad gene, which suggests that E-cad can be used as a predictive indicator of LCBM.

**Table 3:** Emerging biomarkers in LCBM diagnosis and prognosis.

Biomarker	Source	Detection Method	Clinical Utility	Sensitivity/ Specificity	Refs.
S100B	Serum/CSF	ELISA	Predicts BBB disruption	78%/85%	[80,81]
CD44v6	Tumor tissue	IHC	EMT and metastasis prediction	82%/76%	[82]
E-cadherin	Tumor tissue	IHC/Western blot	Loss correlates with BM risk	68%/89%	[83,84]
CSF-ctDNA	Cerebrospinal fluid	NGS/ddPCR	Guides targeted therapy selection	90%/95%	[85,86]
Exosomal miRNA-21	Plasma exosomes	qRT-PCR	Early detection of BM	75%/80%	[87,88]

## 6. Current Status of Treatment of LCBM

**Figure 4:**
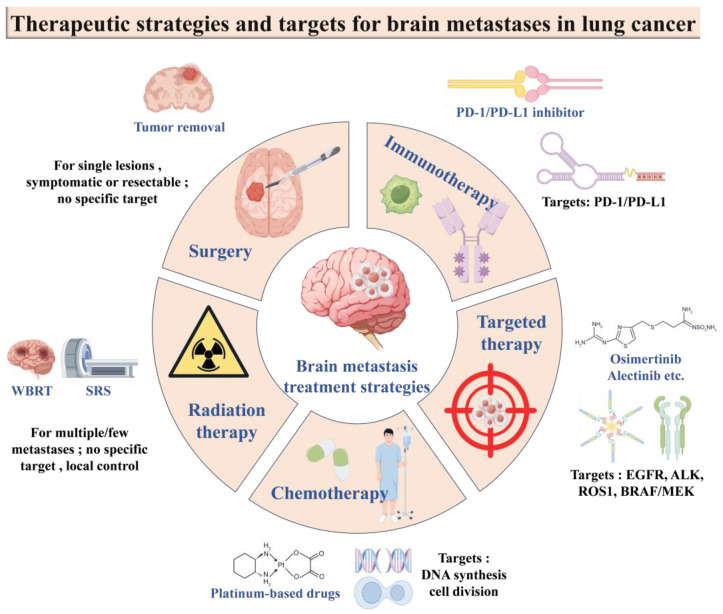
Treatment strategies and molecular targets for lung cancer brain metastases.

After delving deeply into the construction and detection methods of LCBM models, we have further shifted our focus to the current status of clinical treatment for LCBM. Despite the remarkable progress achieved in the diagnosis and treatment of lung cancer in recent years, brain metastasis remains a serious clinical issue that significantly affects the survival rate and quality of life of patients. The incidence of brain metastasis is high, and the prognosis is extremely poor, with a median survival time of only approximately several months to one year. Therefore, the development of effective treatment strategies to improve patients’ prognosis has become the focal point of current research. In recent years, the effects of traditional surgery and radiotherapy have not been satisfactory, and the existence of the BBB also prevents chemotherapy drugs from exerting their efficacy. As scientists continue to explore cancer, targeted therapies and immunotherapy are emerging, and the emergence of new therapies provides new treatment hope for NSCLC patients with brain metastases (Figure 4). Next, we will delve into the diagnostic methods for LCBM, conventional treatment strategies, and the strategies of brain-targeted delivery carriers for small-molecule drugs in detail. Through these discussions, we aim to provide a comprehensive perspective, assisting readers in understanding the current status of LCBM treatment and its future development directions.

### 6.1. Clinical Diagnosis of LCBM

Early and accurate diagnosis of LCBM is crucial for optimizing treatment decisions and improving patient prognosis. Currently, imaging, nanotechnology, liquid biopsy, and other technologies play an important role in the diagnosis of NSCLC brain metastases. With the rapid development of artificial intelligence (AI), deep learning (DL), and other emerging technologies, combined with multimodal image analysis, molecular biomarker detection, and nanoprobe imaging, it is expected to further improve the sensitivity and specificity of NSCLC brain metastasis detection and to promote accurate diagnosis and individualized treatment strategies.

#### 6.1.1. Imaging Joint AI and DL Technologies

Imaging is the cornerstone of LCBM diagnosis, including MRI, computed tomography (CT), and PET. Conventional MRI and CT have limitations in detecting small brain metastases (especially < 5 mm), whereas PET-MRI fusion imaging can provide metabolic and structural information and improve lesion detection rates [89].

In recent years, AI and DL techniques have made significant progress in medical image analysis and have been applied to the automatic detection of lung cancer brain metastases, lesion segmentation, and malignancy prediction. For example, a convolutional neural network (CNN)-based algorithm enables automatic identification of brain metastases and outperforms traditional imaging methods in terms of sensitivity and accuracy [90]. In addition, multimodal images combined with AI analysis (e.g., radiomics + DL modeling) can be used to predict the gene mutation status of brain metastases (e.g., epidermal growth factor receptor (EGFR), anaplastic lymphoma kinase (ALK) mutations), which can provide a basis for precision treatment [91].

With the help of artificial intelligence and deep learning technologies, large amounts of imaging data can be analyzed and diagnosed quickly and accurately. In the future, these technologies are expected to play an important role in the detection of brain metastases, improve the reliability of automated diagnosis, and provide clinicians with more accurate tools to assist decision making.

#### 6.1.2. Nanotechnology

Nanotechnology has shown great potential in the field of tumor diagnosis and treatment, especially in crossing the BBB. Nanoprobe Imaging (NPI) can be used for high-resolution detection of brain metastases. For example, functionalized superparamagnetic iron oxide nanoparticles (SPIONs) can be used as MRI contrast agents to improve the visualization of tiny brain metastases [92]. In addition, nanoprobes such as gold nanoparticles and carbon quantum dots have demonstrated high biocompatibility and trans-BBB capabilities in multimodal imaging techniques such as PET, CT, and fluorescence imaging, which can help in the early detection of brain metastases [93].

In addition to the diagnostic function, the application of nanotechnology in the treatment of brain metastases is equally important. Targeted nanomedicine delivery systems (e.g., lipid nanoparticles, polymer nanocarriers) can be used to deliver brain-targeted chemotherapeutic drugs, overcome the BBB, increase drug concentration, and enhance therapeutic effects [94]. In the future, the “Theranostics” strategy, which integrates nano-imaging and targeted therapies, may become an important development direction for LCBM precision medicine.

#### 6.1.3. Liquid Biopsy Techniques

Liquid biopsy (LB) is a noninvasive, dynamic technique for monitoring tumor progression, mainly through the detection of circulating biomarkers in peripheral blood and cerebrospinal fluid (CSF), including circulating tumor cells (CTCs), circulating tumor DNA (ctDNA), and exosomes [95]. Compared with tissue biopsy, liquid biopsy has the advantages of easy operation and reproducible detection and can be used for early detection of brain metastases, guiding the selection of targeted therapies, evaluating therapeutic response, and prognostic prediction.

CTCs: It has been found that CTC levels are significantly elevated in patients with brain metastatic NSCLC with specific molecular features (e.g., epithelial–mesenchymal transition-related markers) [96]. CTC detection can be used to monitor the risk of brain metastasis and complement diagnostic imaging.

ctDNA: ctDNA analysis in blood or CSF can be used to detect mutations in driver genes, such as EGFR, ALK, and KRAS, to guide targeted therapy decisions. For example, CSF-ctDNA analysis of brain metastatic NSCLC patients reflects the molecular characteristics of intracerebral tumors more accurately than plasma ctDNA [97].

Exosomes and protein biomarkers: Exosomes are nanovesicles released by tumor cells that carry DNA, RNA, proteins, and other information. Studies have shown that exosomal miRNA profiles in patients with brain metastases can be used for early diagnosis and prognostic assessment [98]. In addition, the potential of tumor-associated proteins (e.g., S100B, GFAP) in blood and CSF in the diagnosis of brain metastases is being explored.

In the future, the combination of liquid biopsy and multiomics analysis (e.g., genome, transcriptome, proteome) will promote the development of individualized diagnosis and treatment of NSCLC brain metastases, and provide a more precise biomarker screening strategy for the clinic.

### 6.2. Conventional Treatment Strategies

#### 6.2.1. Surgical Treatment

Surgical treatment of brain metastasis of lung cancer is an important therapeutic tool, which can target brain metastasis on the basis of systemic therapy, with the aim of treating metastatic lesions, improving patients’ symptoms and quality of life, as well as maximizing the survival time of patients. Surgery is mainly applicable to tumors in the brain that are single, in a suitable location, and easily resectable, especially when the tumor or its edema-occupying effect is heavy or leads to hydrocephalus [99]. For multiple brain metastases, if the number of tumors does not exceed three and can be completely resected, surgery can also achieve satisfactory treatment results. In addition, for patients with tumors with a maximum diameter of more than 3 cm, surgery is the treatment of choice; for patients with tumors with a maximum diameter between 1 and 3 cm, the decision should be made based on a comprehensive assessment of the patient’s systemic condition and the risk of surgery. It is worth noting that metastatic tumors located in deep or functional areas of the brain, such as the brainstem, thalamus, and basal ganglia, are not preferred for surgery in principle due to the high rate of surgical disability [100].

#### 6.2.2. Radiotherapy

Radiation therapy for lung cancer brain metastases is an important treatment tool, which includes whole-brain radiotherapy (WBRT) and stereotactic radiotherapy (SRT) (Table 4). WBRT can alleviate neurological symptoms, improve local control of tumors, and provide some control of intracranial subclinical foci in patients with brain metastases from lung cancer [101]. However, due to the dose limitation of normal brain tissue, WBRT is difficult to eradicate intracranial lesions and may delay the emergence of new lesions. SRT, including stereotactic radiosurgery (SRS), fractionated stereotactic radiation therapy (FSRT), and macro segmented stereotactic radiation therapy (HSRT), has the advantages of precise positioning, dose concentration, and relatively small damage, which can well protect the surrounding normal tissues, control local tumor progression, relieve neurological symptoms, and have little effect on neurocognitive function [102,103]. SRT is suitable for the primary treatment of metastases with a single diameter of less than 4–5 cm, the primary treatment of ≤4 metastases, salvage treatment after the failure of WBRT, and adjuvant treatment after resection of intracranial metastases [104]. For patients with multiple brain metastases, close follow-up is required after initial SRT to monitor the occurrence of new intracranial lesions; for large-volume lesions, FSRT is recommended, and its single dose is recommended to be 3.5–4 Gy, with a total dose of 52.5–60 Gy [105]. In addition, for patients who are not suitable for SRS but are expected to have a long survival time, the intensity-modulated radiotherapy technique (IMRT) with WBRT combined with simultaneous dosing of metastatic foci can be used. The efficacy of this treatment modality is superior to WBRT alone, and the difference with SRS is not statistically significant. If enhanced MRI within 1 month of radiotherapy reveals brain metastases >2 cm away from the hippocampus, a hippocampus-protecting simultaneous dosing technique can be used to further protect memory and cognitive function on the basis of improved efficacy.

Notably, with the gradual prolongation of survival time in patients with lung cancer brain metastases, the neurocognitive impairment caused by WBRT, which is mainly manifested as short- and long-term memory loss, reduces patients’ quality of life, which may be related to irradiation-induced hippocampal structural damage [106]. Therefore, several studies have explored WBRT to protect the hippocampus, limiting the maximum irradiation dose in the hippocampal region to 9–16 Gy, which reduces the incidence of neurocognitive decline and the probability of metastasis in the hippocampal region after treatment is only 1.4% to 4.5% [107].

#### 6.2.3. Chemotherapy

Chemotherapy for brain metastasis of lung cancer is an important therapeutic tool, especially for brain metastasis on the basis of systemic therapy. Chemotherapeutic agents such as cisplatin or carboplatin in combination with third-generation cytotoxic analogs and pemetrexed in combination with platinum have been shown to control intracranial lesions in patients with brain metastases from NSCLC, which can result in survival benefit [108]. Specifically, pemetrexed in combination with a platinum-based regimen has shown good efficacy in patients with NSCLC brain metastases. In addition, temozolomide, as an active alkylating agent precursor, is able to cross the BBB and can be applied to improve survival in patients with brain metastases who have previously received WBRT or systemic chemotherapy [109]. However, it is worth noting that chemotherapeutic agents are traditionally considered difficult to penetrate the BBB to exert antitumor effects on intracranial metastatic lesions due to their large molecular weights, charge-carrying properties, and tendency to bind to albumin. Nonetheless, chemotherapy remains an integral part of the comprehensive treatment of patients with NSCLC brain metastases. In practice, the choice and regimen of chemotherapy need to be individualized according to the patient’s specific situation, such as pathological type, genetic status, number and size of brain metastases, and so on. In addition, chemotherapy combined with other treatments, such as radiotherapy, targeted therapy, or immunotherapy, may improve the therapeutic effect. In the course of treatment, it is also necessary to pay close attention to the side effects of chemotherapy and take appropriate symptomatic treatment to alleviate the pain of patients.

#### 6.2.4. Targeted Therapies

Targeted therapies are molecularly designed to target the identified oncogenic gene loci, and the drugs will specifically select the oncogenic loci to combine with the tumor cells and cause-specific death of the tumor cells without affecting the normal tissue cells around the tumor. NSCLC patients should be routinely tested for EGFR, ALK, and other common mutated genes before treatment, and clinical treatment should be carried out after the mutation status is clarified [110]. Currently, EGFR and ALK inhibitors are the main drivers of gene-targeted therapy in the clinic. EGFR inhibitors are small molecules that target the intracellular tyrosine signaling pathway, competitively binding to the tyrosine kinase phosphorylation site in the intracellular segment of the EGFR, blocking the activation of the EGFR by ligands, causing the cell cycle to stall in the G1 phase, promoting apoptosis of tumor cells, and inhibiting tumor growth [111].

#### 6.2.5. Immunotherapy

Immunotherapy is a therapeutic approach to recognize and kill tumor cells by activating or enhancing the body’s own immune system [112]. Immune checkpoint inhibitors (ICI) such as PD-1/PDL-1 inhibitors can restore the immune response of T cells to tumor cells thus achieving the effect of tumor treatment [113]. Currently, these two inhibitors are mainly chosen for immunotherapy in patients with NSCLC brain metastases. The currently approved drugs include PD-1 inhibitors nivolumab, pembrolizumab, and PD-L1 inhibitors Durvalumab, Atezolizumab, and Avelumab. Although immunotherapy will play a pivotal role in the treatment of brain metastases of lung cancer, further research is needed to screen the most suitable population and to prevent and treat adverse effects in the treatment.

### 6.3. Natural Product-Based Drug Delivery Systems for Brain Targeting

Currently, although the therapeutic means for brain tumors include chemotherapy, radiotherapy, molecular-targeted therapy, and immunotherapy, it is difficult to achieve ideal results in the treatment of brain tumors due to the high rates of postoperative recurrence, disability, and morbidity and mortality, the lack of targeting of chemotherapeutic agents, the difficulty in penetrating the BBB, and the susceptibility to multidrug resistance with long-term use. Recent advances in phytomedicine have explored natural compounds (e.g., curcumin, paclitaxel, and ginsenosides) for LCBM therapy. For instance, curcumin-loaded hyaluronic acid/chitosan nanoparticles demonstrated enhanced BBB penetration and anti-glioma effects in preclinical models. Similarly, plant-derived exosome-like nanoparticles (PDENs) show promise as biocompatible carriers for brain-targeted drug delivery.

The BBB is a special structure within the CNS, and its excellent barrier properties can protect the brain from harmful macromolecules and pathogens in the blood circulation, but at the same time, it also restricts the effectiveness of drug delivery [114]. With the continuous deepening of the research on the monomer active ingredients of traditional Chinese medicine (TCM), a variety of TCM active ingredients have shown good brain tumor inhibitory effects in vitro, but most of the drugs have problems such as poor solubility and stability, which lead to low bioavailability after in vivo administration, and the BBB greatly impedes the transcellular transport of drugs from the bloodstream to the brain, limiting their clinical application in the treatment of brain tumors. In order to solve this problem, researchers have developed various drug delivery systems in recent years to increase intracerebral drug delivery. Targeted delivery systems of traditional Chinese medicine can reach the deep brain via blood circulation, increase the concentration and retention time of drugs in the CNS, improve the brain tumor targeting efficiency and therapeutic effect of traditional Chinese medicine, and reduce adverse reactions [115]. According to the different modes of action, TCM-targeted delivery systems can be divided into particle-targeted delivery systems and carrier material surface-modified targeted delivery systems.

#### 6.3.1. Microparticle-Targeted Delivery Systems

The most common particle-targeted delivery systems include liposomes, microemulsions, micelles, and nanoparticles [116] which deliver drugs to the tumor site through the high permeability and retention effect of the tumor.

Among them, liposomes are made from lecithin and ceramide, etc., with bilayer structures, which are widely used in biomedical research because of their good biocompatibility and biosafety [117], and liposomal formulations can improve the water solubility of the drug, enhance drug stability, and carry the drug effectively across the BBB, which is a common carrier for targeting gliomas. The microemulsion is a thermodynamically stable dispersion system spontaneously formed by mixing water, oil, surfactant, and co-surfactant in appropriate proportions, in which the oil-phase component can increase the affinity of the drug to the BBB endothelial cells, which can help the drug to cross the BBB, and it has the advantages of transparency, stability, and perfect absorption; e.g., Shinde et al. [118] made microemulsions from curcumin and docosahexaenoic acid-containing oils, for targeted delivery. Emulsion for targeted delivery of curcumin to the brain site was achieved with good results. The micelles were self-assembled from amphiphilic block copolymers at a concentration greater than the critical micelle concentration, which not only has good biocompatibility, but also improves the solubility and stability of hydrophobic drugs, and the nanoscale micelles can achieve long circulation and tumor tissue targeting [119]; Zheng et al. [120] used biodegradable mono methoxyl poly(ethylene glycol)-poly(propyleneglycol ester) copolymer to formulate curcumin-loaded microemulsions via a self-assembly method by using biodegradable monomethoxy poly(ethylene glycol)-poly(propylenoic acid) copolymers. An assembly method was used to formulate curcumin-loaded nanocolloid micelles, which exhibited stronger therapeutic effects on glioma cells than free curcumin. Nanoparticles are solid colloidal particles with a particle size of 10–1000 nm, which are nanoscale drug delivery systems, i.e., the drug is encapsulated in the skeleton of the carrier material or modified on the surface of the carrier material by covalent linkage adsorption and are widely used in the intracerebral delivery of traditional Chinese medicines; Liu et al. [121] used hyaluronic acid/chitosan as the water-insoluble curcumin carrier and prepared curcumin-loaded hyaluronic acid/chitosan nanoparticles, with the use of free curcumin, which showed stronger therapeutic effects on brain glioma cells than free curcumin. Chitosan nanoparticles and the preparation showed stronger dose-dependent cytotoxicity against C6 glioma cells compared to free curcumin solution. Nano-drugs exhibit broad application prospects due to their advantages in precise targeting, overcoming drug resistance, improving drug properties, and utilizing the tumor microenvironment, making them a research hotspot in the current biomedical field. Although nano-particle drugs specifically targeting brain metastases are still in the preclinical research stage, relevant studies have already achieved positive progress. For instance, The HER3 ligand-mimicking nanobioparticles designed by the team led by Lali K. Medina-Kauwe [122] have demonstrated outstanding advantages in preclinical studies. The team utilized organoid chips derived from induced pluripotent stem cells to mimic the humanized blood–brain barrier, conducting in-depth research on the penetration mechanism of the nanobioparticles. The research indicates that these particles can accurately recognize and bind to the HER3 receptors on the surfaces of both the blood–brain barrier and brain tumor cells. With their unique structures and surface modifications, they can efficiently penetrate the blood–brain barrier and specifically accumulate in brain tumors. This achievement has laid a solid foundation for the subsequent initiation of human clinical trials. It is expected that these nanobioparticles will enter the clinical treatment stage in the near future, bringing new treatment options for patients with brain tumors. In addition, another study has developed a P-selectin-targeted nanocarrier [123]. This nanocarrier can specifically bind to P-selectin on the surface of endothelial cells of the blood–brain barrier, inducing a caveolin-1-dependent transcytosis, thereby achieving active crossing of the blood–brain barrier. This mechanism provides a new pathway for the delivery of drugs to the brain, and it is expected to solve the problem that traditional drugs have difficulty effectively penetrating the blood–brain barrier. It offers new drug delivery strategies and research directions for the treatment of brain diseases, such as brain tumors and neurodegenerative diseases, and has potential application value in the clinical treatment of brain diseases. These studies all highlight the enormous potential of nanodrugs in the treatment of brain metastases. With the deepening of research, nanoparticles are expected to bring about new changes in the treatment of brain tumors and improve the prognosis of patients.

#### 6.3.2. Targeted Delivery Systems for Surface Modification of Carrier Materials

Due to the obvious limitations of the particle-targeted delivery system, which lacks a high degree of selectivity and affinity for tumor cells, the drug often fails to accurately reach the tumor cells during its in vivo transport, leading to the distribution of part of the drug in non-target tissues, which wastes the drug resources and may also trigger unnecessary adverse reactions [124]. In order to break through these bottlenecks, surface-modified targeted delivery systems for carrier materials have emerged.

Based on the high-affinity property between ligand and receptor, this system cleverly exploits the differences in receptor or antigen expression on the surface of tumor cells and normal cells to achieve higher trans-BBB transporter ability or tumor penetration. For example, certain tumor cell surfaces overexpress specific receptors, such as EGFR, transferrin receptor, etc., while the expression on the surface of normal cells is relatively low. By modifying specific ligands targeting these receptors on the surface of the carrier material, when the carrier enters the body, it is capable of actively recognizing and binding to the receptors on the surface of the tumor cells, thus realizing the selective targeting delivery of drugs [125]. This not only increases the concentration of the drug at the tumor site and enhances the anti-tumor effect, but also reduces the damage to normal tissues.

Among the common active targeting carriers, small molecules have become a common choice for surface modification due to their simple structure, easy synthesis, and good stability. For example, folic acid, as a small molecule ligand that specifically binds to folate receptors overexpressed on the surface of tumor cells, has been widely used in the modification of carrier materials for targeted therapy of brain tumors and other tumors [126]. Proteins are highly specific and biologically active, such as antibodies, which can accurately recognize antigens on the surface of tumor cells, but their preparation is complex, costly, and problematic in terms of immunogenicity. Peptides, on the other hand, combine some of the advantages of small molecules and proteins; they have relatively simple structures and high affinity, are easy to synthesize and modify, and show great potential in the field of carrier surface modification. For example, membrane-penetrating peptides can help carriers cross biological membranes, including the BBB, and facilitate drug entry into brain tumor tissues; cell-penetrating peptides can not only improve the transmembrane ability of carriers but also enhance the uptake of drugs in cells [127].

With the continuous deepening of research, the application of carrier material surface modification targeted delivery systems in brain tumor therapy is becoming more and more promising. In the future, it is expected that by further optimizing the design, screening, and synthesis of carrier materials, more efficient, safer, and precisely targeted delivery systems will be developed, which will bring new therapeutic hope for brain tumor patients and promote the development of the whole cancer treatment field in the direction of more accurate and personalized.

**Table 4:** Therapeutic strategies for LCBM: mechanisms and clinical outcomes.

Therapy	Mechanism	Advantages	Limitations	Median Survival Benefit	Key Clinical Trials/Studies
WBRT	Targets all CNS lesions via ionizing radiation	Rapid symptom relief	Neurocognitive decline	3–6 months	QUARTZ Trial [128]
SRS	Precise high-dose radiation to focal lesions	Sparing of healthy tissue	Limited to ≤4 lesions	10–12 months	N107C/CEC.3 Trial [129]
EGFR-TKIs	Inhibits EGFR-driven tumor growth	BBB penetration, systemic control	Resistance mutations (e.g., T790M/C797S)	15–20 months	AURA3 Trial [130]
ICI	Blocks PD-1/PD-L1 interaction	Durable responses	Low CNS penetration	8–12 months	KEYNOTE-189 [131]
Nanoparticle Drug Delivery	Enhances BBB permeability via ligand targeting	Improved drug accumulation in brain	Toxicity of carrier materials	Under investigation	Preclinical studies (e.g., SPIONs for MRI-guided delivery) [132]

## 7. Conclusions

Animal modeling is an important means and platform for experimental research [129], and it is of great significance for the study of brain metastasis of lung cancer in terms of its occurrence mechanism, diagnosis, and treatment. Each of the five modeling methods mentioned in this paper has its own advantages and disadvantages, among which intracerebral injection modeling has a high success rate, but it cannot mimic the process of tumor cells passing through the BBB and thus colonizing the brain after forming from the in situ position, which is limiting in the study of the mechanism of tumor brain metastasis, but it can be used for the pharmacodynamic evaluation of antitumor drugs for brain tumors. Left ventricular injection can well mimic the tumor cell metastasis through the bloodstream, which greatly reduces the capture of tumor cells in the pulmonary capillary bed and increases the number of cells entering the circulation, thus improving the success rate of modeling. Moreover, this model is in line with the biological characteristics of brain metastasis, and the pathomorphology and imaging manifestations of metastatic foci formed are more similar to those of the clinical ones, which makes this modeling method simple and with a higher success rate. Carotid artery injection can well simulate the process of bloodstream metastasis of lung cancer, with high brain specificity, which can simulate the process of tumor cells from entering the circulation, capillary stagnation, crossing the BBB, and ultimately colonizing and growing in brain tissues, which increases the number of tumor cells passing through the brain parenchyma, prolongs the time for tumor cells to grow and proliferate in the brain, and results in a higher success rate of modeling [133]. However, this modeling method requires a high level of skill from the experimenter, who needs to be familiar with the anatomical relationship of the carotid arteries and have certain surgical skills. Tumor cells injected into the tail vein will first be retained in the lungs and have a high probability of metastasis in other parts of the body such as lungs and bones, which is likely to cause mice to die of malignant stasis in advance, and only a few tumor cells can enter the body circulation, which may then form brain metastasis foci, with a low success rate of establishing brain metastasis models. In situ injection better simulates the process of tumor occurrence, development, and metastasis, but it is prone to thoracic extensive planting, the experimental cycle is long and the brain metastasis rate is low, often needs to be repeated in the experimental animal body before there is a low probability of inducing brain metastasis, the growth consistency of the animal model is poor, and it is not easy to form a control.

Clinical brain metastases are difficult to treat and have a poor prognosis, and the development of animal models of brain metastases is an important support for LCBM treatment research. The brain metastasis models constructed by different methods focus on reflecting the different aspects of metastatic tumor development, and the research on brain metastasis of lung cancer should be based on the characteristics of the model, the characteristics of the lung cancer itself, and the actual needs to choose the appropriate model construction method.

## Figures and Tables

**Table 1:** Scope of application and advantages and disadvantages of common animal models of LCBM.

Modeling Method	Applicable Research Scope	Advantages	Disadvantages
Stereotactic Intracranial Injection	Suitable for pharmacological evaluation of anti-brain tumor drugs	The high success rate in the model establishment	Cannot simulate the process of tumor cells forming in situ and then colonizing in the brain through the BBB
Tail Vein Injection	It better simulates the various processes of tumor development, progression, and metastasis, and is suitable for research on the occurrence mechanism, diagnosis, and treatment of brain metastasis of lung cancer	Simple operation, high likelihood of invasion and metastasis	Low brain specificity, prone to metastasis in other sites causing premature death in mice
In situ Injection		High consistency with clinical lung cancer, high tumor formation rate, and ability to metastasize	High technical difficulty in operation, prone to causing pneumothorax and death in mice, low brain metastasis rate, and short survival time
Left Ventricular Injection		Consistent with the biological characteristics of brain metastasis, high success rate in model establishment	High surgical difficulty, prone to causing death in mice during model establishment
Carotid Injection		Well simulates the hematogenous metastasis process of lung cancer, high brain specificity, and higher success rate in model establishment	High surgical skill requirement, need to be familiar with the anatomical relationship of the carotid artery

## Data Availability

Not applicable.
